# A Comparative Study on Three Different Transducers for the Measurement of Nonlinear Solitary Waves

**DOI:** 10.3390/s130101231

**Published:** 2013-01-18

**Authors:** Xianglei Ni, Luyao Cai, Piervincenzo Rizzo

**Affiliations:** Laboratory for Nondestructive Evaluation and Structural Health Monitoring Studies, Department of Civil and Environmental Engineering, University of Pittsburgh, 3700 O'Hara Street, Pittsburgh, PA 15261, USA; E-Mails: xin1@pitt.edu (X.N.); luc21@pitt.edu (L.C.)

**Keywords:** highly nonlinear solitary waves, discrete particle model, transducers, magnetostrictive sensors

## Abstract

In the last decade there has been an increasing interest in the use of highly- and weakly- nonlinear solitary waves in engineering and physics. Nonlinear solitary waves can form and travel in nonlinear systems such as one-dimensional chains of particles, where they are conventionally generated by the mechanical impact of a striker and are measured either by using thin transducers embedded in between two half-particles or by a force sensor placed at the chain's base. These waves have a constant spatial wavelength and their speed, amplitude, and duration can be tuned by modifying the particles' material or size, or the velocity of the striker. In this paper we propose two alternative sensing configurations for the measurements of solitary waves propagating in a chain of spherical particles. One configuration uses piezo rods placed in the chain while the other exploits the magnetostrictive property of ferromagnetic materials. The accuracy of these two sensing systems on the measurement of the solitary wave's characteristics is assessed by comparing experimental data to the numerical prediction of a discrete particle model and to the experimental measurements obtained by means of a conventional transducer. The results show very good agreement and the advantages and limitations of the new sensors are discussed.

## 1. Introduction

In the last fifteen years the numerical and experimental studies on the propagation of nonlinear solitary waves in one-dimensional chains of granular media, and in particular of spherical elastic beads, have thrived [1–20]. The nonlinearity arises from the Hertzian type contact between two adjacent particles and zero tensile force. When the chain is non- or weakly-compressed by means of its self-weight or by the action of some form of static pre-compression, highly nonlinear solitary waves (HNSWs) can form and propagate in the chain. The term “weakly” implies that the pre-compression is very small compared to the dynamic force amplitude associated with the wave propagation.

It has been demonstrated that HNSWs propagating in granular crystals have the potential to be used as acoustic lenses [21], vibration absorbers [22], impurity detectors [15,23], acoustic diodes [24] and as a tool for nondestructive testing [25–28]. In some of these engineering applications, the measurement of the dynamic force is necessary. To date, this measurement is attained either by means of one or more sensor beads placed in the chain [6–14,25–30] or by using a force sensor mounted at the base of the chain [4–8]. The former usually consists of two half beads bonded to a thin piezoelectric crystal in order to form a sensor particle able to measure the dynamic force at its center. The main advantage of this assembly is two-fold: it can be placed anywhere in the chain; it does not alter the characteristics of the propagating wave, since the geometry and the material property of the sensor are essentially identical to those of the other particles composing the chain. However, the manufacture of sensor beads requires machining and therefore can be time-consuming and costly. Moreover, once in place, the bead should not be allowed to rotate in order to maintain its sensitivity constant and the wires are prone to accidental breakages. A force sensor instead measures the characteristics of the wave at the end of the chain. This configuration is unpractical when the same end needs to be in contact with another material or structure. Finally, few studies exploited the photoelasticity to measure the stress wave propagation in photoelastic grains [31,32]. However, this approach is not suitable for metallic or other non-photoelastic materials and might be expensive.

In the study presented here, we investigated numerically and experimentally two alternative sensing systems to measure the propagation of solitary waves in a 1-D chain of metallic particles. The first design replaces the sensor beads with piezo rods having thickness and diameter comparable to the size of the particles composing the chain. The second system considers the use of coils wrapped around a segment of the chain to create a magnetostrictive sensor (MsS). To the best of the authors' knowledge, the use of magnetostriction or piezoelectric cylinders to measure the propagation of HNSWs was never reported in the past. In this paper the working principles of these novel transducers are introduced and the experimental results are compared to the measurements obtained using conventional instrumented beads and to the numerical prediction derived with a discrete particle model.

The paper is organized as follows: the experimental setup is described in Section 2. The principles of the three types of sensors are introduced in Section 3. Section 4 presents the numerical model of wave propagation in a chain of spherical particles. In Section 5, the experimental results are presented. Finally, Section 6 concludes the paper with a discussion on the advantages and disadvantages of the three sensing configurations.

## 2. Experimental Setup

**Figure 1.**
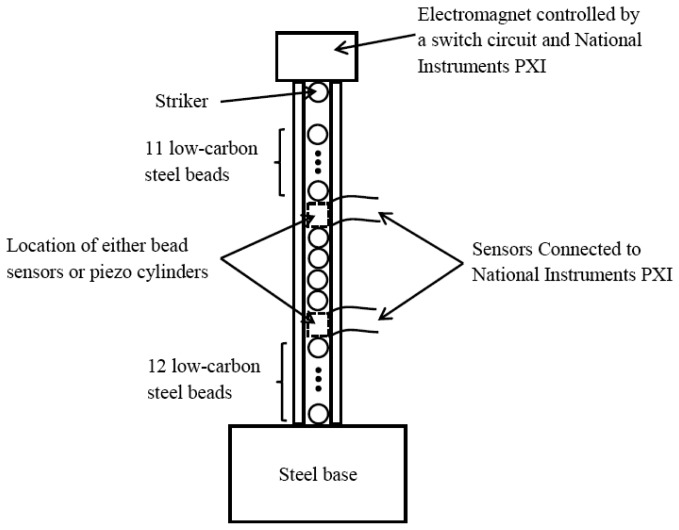
Schematic diagram of the experimental setup.

In order to compare the novel sensing systems to the conventional one, a plastic tube with inner diameter of 4.8 mm and outer diameter of 12.7 mm was filled with twenty nine 4.76 mm-diameter, 0.45 gr, low carbon steel beads (McMaster-Carr product number 96455K51). An identical bead was used as striker. For convenience, the particles are herein numbered 1 to 30 where particle 1 identifies the striker and particle 30 represents the sphere at the opposite end of the chain. The stroke of the particle 1, equal to 7.2 mm, was governed by an electromagnet mounted on top of the tube and remotely controlled by a switch circuit connected to a National Instruments PXI running in LabVIEW. Figure 1 schematizes the setup described above.

**Figure 2.**
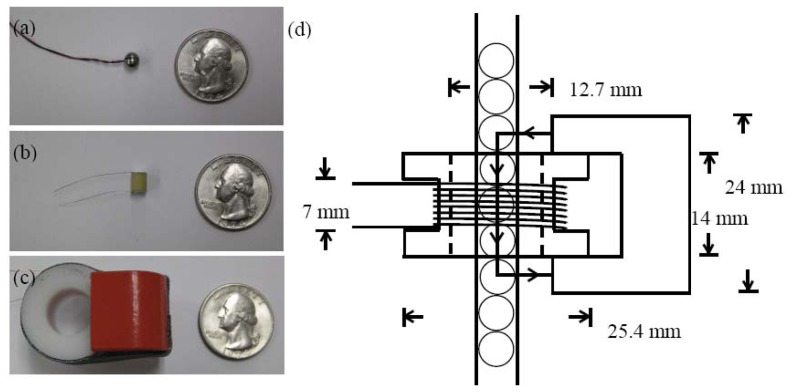
Sensing technologies used in this study. (**a**) Bead sensor formed by a thin piezoelectric crystal embedded between two half particles, (**b**) commercial piezo rod, (**c**) magnetostrictive sensor formed by a coil and a bridge magnet, (**d**) Schematic diagram of one magnetostrictive sensor assembled with the tube filled with spherical particles.

Three pairs of sensors were used in this study: bead sensors, rod-form piezos, and MsSs. Each bead sensor was assembled by embedding a zirconate titanate based piezogauge (3 mm by 3 mm by 0.5 mm) inside two half steel spheres, as shown in Figure 2(a). They were located at the positions 13 and 18 in the tube. Figure 2(b) shows instead one of the two piezoelectric cylinders. They were custom made (Piezo Kinetics Inc. ND0.187-0.000-0.236-509) with 36AWG × 25.4 mm soldered tinned copper lead wires. The rods had nominal dimension 4.76 mm outer diameter and 6 mm height. According to the manufacturer, their mass was 0.8144 g, Young's modulus 63 GPa, and Poisson's ratio equal to 0.31. When they were used, the piezo cylinders replaced the bead sensors at location 13 and 18 in the tube. Finally, each MsS consisted of a 7 mm coil made of AWG36 magnetic wire and 1100 turns wrapped around a plastic tube having inner diameter of 12.7 mm and 1.6 mm thick. A permanent bridge magnet (McMaster-Carr product number 5841K12) was fixed to the coil as shown in Figure 2(c) in order to create a constant magnetic field parallel to the longitudinal axis of the chain. Figure 2(d) shows the schematic of the sensor once the plastic tube containing the chain of particles was inserted. The two magnetostrictive transducers were mounted such that their centers were located at the same elevation of particles 13 and 18.

The capability and the repeatability to measure the amplitude and speed of the HNSWs was evaluated by taking 500 measurements at 10 MHz sampling rate for each sensing configuration.

## 3. Background

In a one-dimensional chain of spherical particles, the interaction between two adjacent beads is governed by the Hertz's law [1,4]:

(1) $$F=A\delta^{3/2}$$

where *F* is the compression force between two beads, *δ* is the closest approach of particle centers, and *A* is a coefficient given by:

(2) $$A=\frac{E\sqrt{2R}}{3\left(1-\nu^{2}\right)}$$

where *R* is the radius of the particles, and ν and *E* are the Poisson's ratio and Young's modulus of the material constituting the particles, respectively. The combination of this nonlinear contact interaction and a zero tensile force in the chain of spheres leads to the formation and propagation of compact solitary waves. More detailed formulation on the analytical foundation of HNSW propagation may be found in several references [1–12].

A single pulse is commonly induced by mechanically impacting the first bead of the chain with a striker having the same mass of the particles composing the chain. Ni *et al.* [13] showed that laser-pulses can also be used in lieu of mechanical impacts. When a bead sensor is inserted in a chain, the force measured is the average of the dynamic forces at the bead's ends [6]. Similarly, the force measured by a piezoelectric cylinder is the average of the dynamic forces at the two cross-section ends. Both sensing configurations are based on the use of piezoelectric crystals.

The MsS takes advantage of the efficient coupling between the elastic and magnetic states of the ferromagnetic particles and in particular of the magnetostrictive phenomena that convert magnetic energy into mechanical energy and vice versa [33]. The magnetostriction principle can be used in the active or in the passive mode based upon the Faraday's law and the Villari's effect, respectively. According to the Faraday's law an electrical current passing along a coil induces a magnetic field that is perpendicular to the current's direction. If the coil enwinds a ferromagnetic material, an alternating current passing through a wire creates a time-varying magnetic field within the coil that, in turn, produces a change of magnetostriction of the material. The subsequent deformation known as Joule's effect [34] produces a stress wave. According to the Faraday's law the voltage output in the coil can be expressed as [33,35–37]:

(3) $$V=-N\frac{d\varphi }{dt}$$

where *V* is the induced voltage in the coil, *N* is the number of turns of the coil, *φ* = *BS_c_* is the magnetic flux. Here *B* is the magnetic induction, *S_c_* is the area of coil in the magnetic field. Equation (3) can be written as:

(4) $$V=-NS_{c}\frac{dB}{dt}$$

The inverse mechanism can be used for the detection of waves. A pulse propagating in the ferromagnetic material modulates an existing magnetic field by means of the Villari's effect [33,38], thereby exciting a voltage pulse in the receiver coil. In both transduction and detection, a constant magnetic field (bias) is superimposed to enhance the coupling between the elastic and magnetic states, *i.e.*, to increase the signal-to-noise ratio of the stress wave generated and detected by the magnetostrictive transducer. One of the authors have designed, built, and used magnetostrictive transducers for the generation and detection of ultrasonic guided waves in strands, solid cylinders, and pipes [39–44].

In the design of the MsS used in the present study we exploited the Villari effect to detect the propagation of nonlinear solitary waves across the chain. The particles are the magnetostrictive material subjected to a biased magnetic field and are surrounded by a coil. We hypothesized that the change of the magnetic induction is proportional to the change of the dynamic contact force between neighboring particles. The output voltage was proportional to the time-derivative of dynamic contact force:

(5) $$V\propto \frac{dF}{dt}$$

Therefore, the dynamic force associated with the solitary wave propagation is proportional to the integral of the sensor output voltage. Based upon the geometry of the MsS [see Figure 2(d)], we assumed that the permanent magnet biased four contact points, which implied that the dynamic force measured by the coils sensor could be reasonably considered the average of four dynamic forces at these points.

## 4. Numerical Model

### 4.1. Formulation

**Figure 3.**
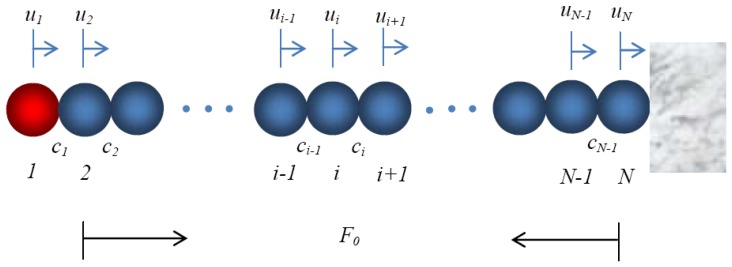
Schematic diagram of the one-dimensional discrete element model. The *c_1_, c_2_*, …, *c_N_* indicates the points of contact between two neighboring particles. When the presence of the piezo rod is modeled, the spheres 13 and 18 are replaced by solid rods.

The experimental setup was simulated using a chain of spherical particles in contact with a wall which was considered as a half-infinite medium, as shown in Figure 3. We adopted the discrete particle model [1,11] to predict the characteristics of the nonlinear solitary pulses generated by the impact of a striker. In the model the motion of the particles was considered in the axial direction, and the interaction between two adjacent spheres was governed by the Hertz's law (Equation (1)). The equation of motion of the *i-th* particle can be expressed as [1,11,12]:

(6) $$\begin{array}{ccc} m_{i}{\ddot{u}}_{i}\;=\;A_{i-1}\delta_{i-1}^{3/2}-A_{i}\delta_{i}^{3/2}+\gamma_{i-1}{\dot{\delta }}_{i-1}-\gamma_{i}{\dot{\delta }}_{i}+F_{i} & , & i=1,2,\ldots ,N \end{array}$$

Where:

(7) $$A_{i}=\{\begin{array}{ccc} 0 & , & i=0\\ A_{c}=\frac{E\sqrt{2R}}{3(1-\nu^{2})} & , & i=1,2,\ldots ,N-1\\ A_{w}=\frac{4\sqrt{R}}{3}{\left(\frac{1-\nu^{2}}{E}+\frac{1-\nu_{w}^{2}}{E_{w}}\right)}^{-1} & , & i=N \end{array}$$

(8) $$\gamma_{i}=\{\begin{array}{ccc} 0 & , & i=0\\ \gamma_{c} & , & i=1,2,\ldots ,N-1\\ \gamma_{w} & , & i=N \end{array}$$

(9) $$\delta_{i}=\{\begin{array}{ccc} {\left[-u_{1}\right]}_{+} & , & i=0\\ {\left[u_{i}-u_{i+1}\right]}_{+} & , & i=1,2,\ldots ,N-1\\ {\left[u_{N}\right]}_{+} & , & i=N \end{array}$$

Here, the subscripts *c* and *w* refer to the point of contact between two neighboring particles and the point of contact between the last particle and the half-infinite wall, respectively. The values of *R*, *m*, and *u* are respectively the radius, mass, and axial displacement from the equilibrium position of the particle. *A* is the contact stiffness between adjacent beads (*A_c_*) or between the last bead and the wall (*A_w_*). *γ* is a coefficient that takes into account the dissipative effects associated with the contact of the chain with the inner tube's surface and the wall [11,12]. *F* is the sum of the body force, e.g., gravity, and external forces applied on the chain. The dot represents the time-derivative while the operator []_+_ returns the value of the variable if the variable is positive, otherwise it returns 0. Finally, *E* and ν are the Young's Modulus and the Poisson's ratio, respectively, of the beads (*E_c_*, *ν_c_*) and of the wall (*E_w_*, *ν_w_*).

The impact of the striker was simulated by setting the initial displacement *u_0_*=*0* and the initial velocity ${\dot{u}}_{0}=\sqrt{2gh}$, where *g* is the gravitational constant and *h* is the experimental falling height (*h* = 7.2 mm). The other initial conditions were *u_i_* = 0 for *i* = *1, 2*, …, *N*, *u̇*_1_ = *u̇*_0_ and *u̇_i_* = 0 for *i* = *1, 2*, …, *N*. The differential Equation (6) was solved to calculate *u* and *u̇* for each particle by using the fourth order Runge-Kutta method.

The model of the chain with the instrumented beads did not consider the fact that they are slightly heavier than the other spheres. This is because it was demonstrated [6,8] that the effect of the mass difference on the wave propagation can be neglected in the model. As such, particles 13 and 18 had the same mass of all the other particles. Moreover, because the piezogauge-half sphere contact stiffness is much higher than the sphere-sphere contact stiffness, the sensor still could be considered as a rigid body.

In order to model the presence of the piezo rods, the modeled chain comprised two solid rods having the same geometric and material properties of the piezoelectric cylinders at position 13 and 18. And the mass and contact stiffness (terms *A_i_*) at positions 13 and 18 were replaced accordingly. Finally, the model relative to the presence of the coil and the bias magnet, considered the tube filled with 29 particles. The presence of the magnetic bias was included by considering four particles per coils subjected to a static compressive force equal to 1.8 N. This value was estimated by comparing the experimental wave speed of the incident solitary wave to the analytical prediction provided by the long-wavelength limit [1,7,13]. Irrespective of the sensing system considered the model included the static pre-compression due to the self-weight of the particles, and the dissipation coefficients *γ*, whose determination will be discussed in Section 5.1.

### 4.2. Numerical Results

**Figure 4.**
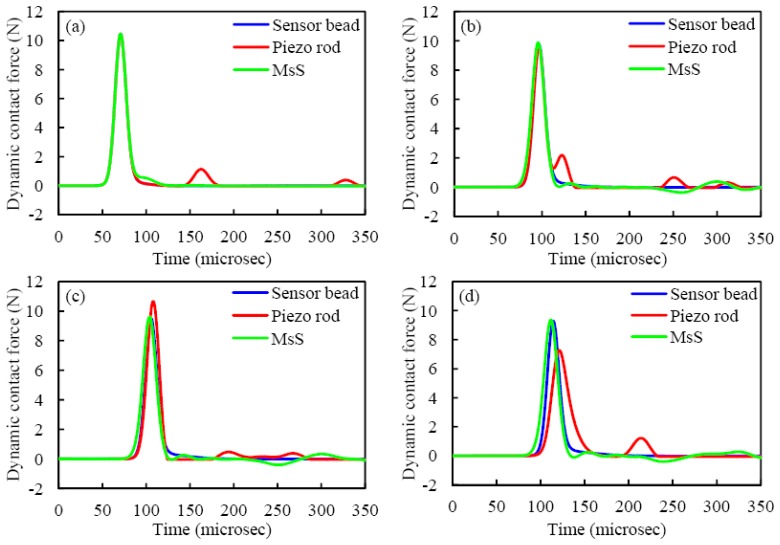
Discrete particle model results showing the temporal force profile for all threesensing configurations at contact points: (**a**) *c_8_*, (**b**) *c_11_*, (**c**) *c_12_*, and (**d**) *c_13_*.

The numerical model was applied to simulate the three setups described in Section 2. To predict the measurements of the three types of transducers, the numerical values of the force-time profiles at contact points c_8_, c_11_, c_12_, and c_13_ (see the notation in Figure 3) are presented in Figure 4. The figure shows the presence of a single solitary wave, whose amplitude and time of arrival are almost the same for all three types of sensors. The pulse measured by MsS at contact points c_11_-c_13_ has a slightly earlier arrival due to the presence of the magnetically induced precompression. The presence of the cylinder alters the temporal force profile due to the following mechanism. As the single pulse propagates, the particle 11 compresses the next particle giving rise to the first peak visible in Figure 4(b). Because the piezo rod is heavier than particle 12, particle 12 bounces back. Thus, the corresponding amplitude of the dynamic force is higher and it is visible in Figure 4(c). By bouncing back, particle 12 gets closer to sphere 11 originating the small hump visible in Figure 4(b). This reflected wave propagates backward to the top of the chain and it appears also in Figure 4(a) at around 170 microseconds. Meantime, after particle 12 is compressed by particle 11 for the second time, its velocity becomes slightly larger than that of the cylinder. This compresses again particle 12 against the rod, as demonstrated by the small pulse in Figure 4(c) at 200 microseconds. This creates a state of compression between the cylinder and its neighboring particle 14, which gives rise to a secondary pulse that trails the incident wave and is visible in Figure 4(d) at 220 microseconds. The figure also indicates that the amplitude of the wave passing through the cylindrical sensor reduces significantly when compared to the same pulse monitored by the bead sensor or the MsS.

**Figure 5.**
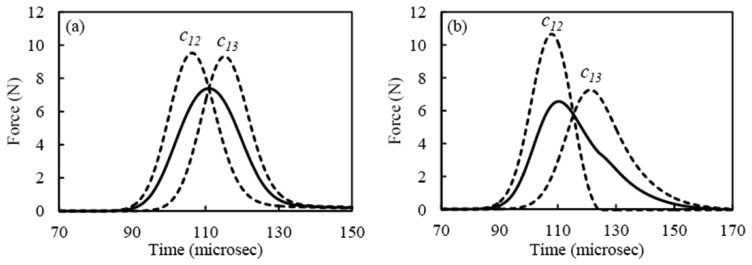

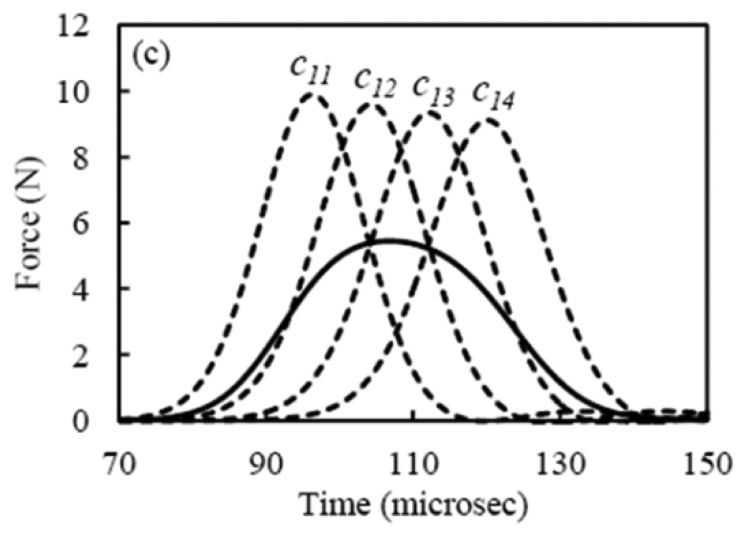
Discrete particle model results showing the temporal force profile at some contact points (dashed lines) and as measured by three sensors (solid lines): (**a**) bead sensor, (**b**) piezo rod, (**c**) magnetostrictive sensor.

The numerical results of the force profiles at position 13 for the three sensing systems are shown in Figure 5. Figure 5(a) refers to the sensor bead. The dashed lines represent the dynamic forces at the contact points c_12_ and c_13_, whereas the continuous line is the average value of the two dynamic contact forces and it represents the force measured by the sensor bead [6]. Figure 5(b) presents the results relative to the rod. Similar to Figure 5(a), Figure 5(b) shows the values of the force at the contact points of the cylinder with the particles 12 and 14 (dashed lines) and the averaged value (continuous line). Because the cylinder's mass and stiffness are different than those of the spheres, the incoming wave is partially reflected at the interface, thus reducing the amplitude of the transmitted pulse.

Finally, Figure 5(c) shows the results associated with the presence of the coil centered at location 13. Owing to the length of the coil and the position of the magnetic bias (Figure 2(d)) the dynamic forces at contact pointsc_11_ to c_14_ are presented. Because the force measured by the MsS is the average of four dynamic contact forces at contact points 11 to 14, its amplitude is smaller and its duration is longer with respect to the dynamic force measured by the other two sensing systems.

## 5. Experimental Results and Discussions

**Figure 6.**
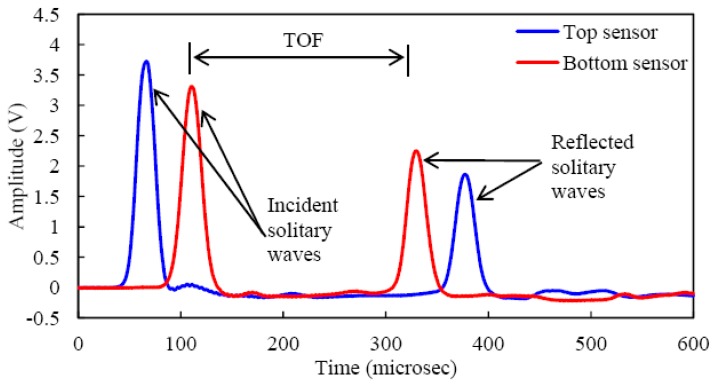
Typical waveforms measured by the bead sensors.

Although the dissipation coefficients can be determined empirically by measuring the magnitudes of the dynamic forces at different positions in the chain [11,12], we adopted another approach that is illustrated here. Figure 6 shows the voltage output measured by both spherical sensors. The first pulses represent the main solitary wave traveling from the impact point to the wall, while the second pulses are the waves reflected from the rigid wall. We characterized the HNSW by means of three parameters: the time-of-flight (TOF), the speed, and the amplitude ratio (AR). The first parameter denotes the difference in the transit time at a given position between the incident and the reflected wave. The speed can be simply computed by dividing the distance between two sensors over the difference in the arrival time of the two amplitude peaks. Finally, we define the AR as the ratio of the reflect wave amplitude over the incident pulse amplitude.

### 5.1. Dissipation Coefficients

In order to calibrate the numerical model to our experimental setup, the dissipation was taken into account. Because the force amplitudes of both incident and reflected solitary pulses were proportional to the voltage-force conversion factor, their AR was independent upon this conversion factor. We computed the dissipation coefficient by considering the 500 experimental measurements of the amplitude ratios *AR_top_* and *AR_bottom_* measured by the sensors located at positions 13 and 18, respectively. For different combination of dissipation coefficients *γ_c_* and *γ_w_*, the numerically predicted *AR_top-num_* and *AR_bottom-num_* were calculated. Then an objective function *y*:

(10) $$y(\gamma_{c},\gamma_{w})=\text{norm}\left(\left[\frac{AR_{\text{top}-\text{num}}-AR_{\text{top}}}{AR_{\text{top}}},\frac{AR_{\text{bottom}-\text{num}}-AR_{\text{bottom}}}{AR_{\text{bottom}}}\right]\right)$$

was defined in terms of the difference between numerical and experimental results. We optimized this function by finding the combination of *γ_c_* and *γ_w_* that minimized the value of the objective function subjected to the following constraints: *γ_c_* ≥ 0 and *γ_w_* ≥ 0. The result of the optimization process yielded to *γ_c_*= 4.8 kg/s and *γ_w_* = 0. As it is not very plausible that there is no dissipation at the wall, the result of the optimization may suggest that the dissipation along the chain might be more dominant than the energy loss from the last particle-wall interaction.

As is said earlier, when the piezoelectric cylinders and MsSs were used, the sensor beads were replaced by the two piezo rods and two spheres, respectively. Because the coefficient *γ_c_* accounts for the dissipation along the entire chain, we assumed that the replacement of two particles out of 29 had negligible effect on the value of *γ_c_*. Thus, the value *γ_c_* = 4.8 kg/s was applied to all three kinds of sensing technology.

### 5.2. Voltage-Newton Conversion

**Figure 7.**
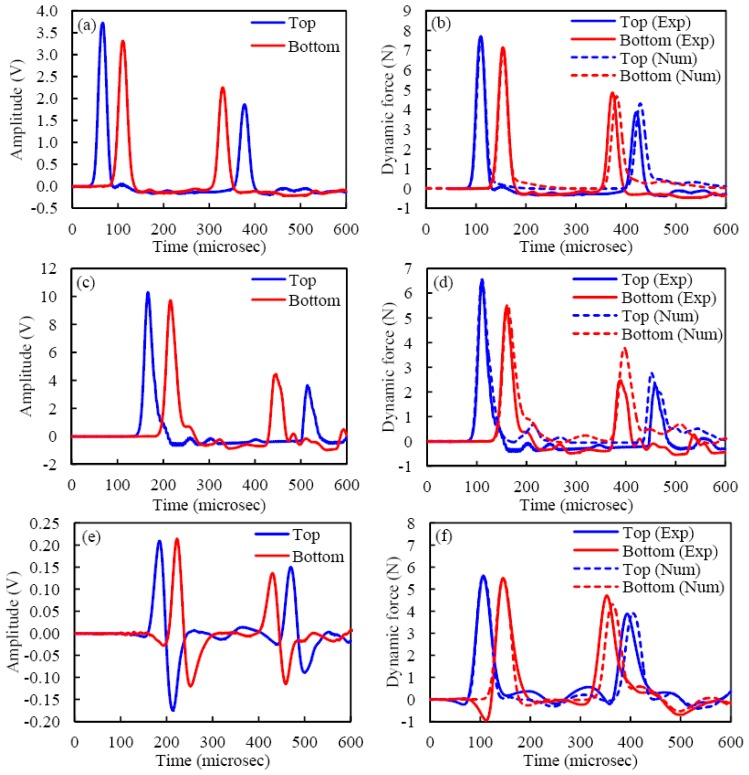
(**a**) Experimental results for bead sensors, (**b**) comparison of experimental and numerical results for bead sensors, (**c**) experimental results for cylindrical sensors, (**d**) comparison of experimental and numerical results for cylindrical sensors, (**e**) experimental results formagnetostrictive sensors, (**f**) comparison of experimental and numerical results for magnetostrictive sensors.

In applications such as nondestructive testing, voltage measurements are sufficient to correlate the characteristics of the solitary pulses to the properties of the structure or material under inspection. However, other engineering applications may require the quantitative measurement of the dynamic force associated with the traveling pulse. Thus, the relationship between this force and the output voltage from the sensor needs to be known. To establish this relationship, we adopted the following procedure for all sensing configuration. The experimental time profiles, expressed in Volts and collected using the experimental setup described in Section 2 were compared to the force profiles (expressed in Newton) computed with the discrete particle model described in Section 4. The model considered the effect of dissipation. Figure 7(a,c,e) shows the voltage output associated with the three transducers pair. One out of 500 measurements is displayed. With respect to Figure 7(a,c), Figure 7(e) shows negative values of the output voltage. When the solitary pulse travels through the particles surrounded by the MsS, there is an increase in compression due to the contribution of the dynamic contact force. This increase generates a positive gradient of the magnetic flux visible in the positive output voltage. When the pulse propagates away, the compression between adjacent spheres decreases, creating a negative gradient of the magnetic flux. This negative gradient is represented by the negative portion of the signal in Figure 7(e). Similarly, the dashed lines in Figure 7(b,d,f) shows the corresponding numerical predictions. Because the overall shapes of the incident experimental and numerical temporal profiles were almost identical, a conversion factor *K_i_* associated with each measurement *i* was calculated as:

(11) $$K_{i}=\frac{F_{\text{Num}}}{V_{i,\mathrm{Exp}}}\;(i=1,2,\ldots ,500)$$

Here *V_i,Exp_* is the maximum experimental output voltage, and *F_Num_* is the maximum amplitude of the dynamic force determined numerically. A unique conversion factor *K* was then established by averaging 500 ratios. Table 1 summarizes the coefficients determined for every transducer. The small standard deviations prove the repeatability of the setup and the consistency of the novel sensing systems. It is noted that, as the force measured by the MsS is related to the integral of the voltage (see Equation (5)), the corresponding units for factor *K* are different from the others.

### 5.3. Comparison of the Numerical and Experimental Results

In the last part of our study, we quantitatively compared the shape of the experimental and numerical force profiles. Figure 7(b) shows the time series associated with the bead sensors. For convenience of representation, the experimental data are shifted horizontally in order to overlap the numerical and experimental peak of the incident pulse at the particle 13. Clearly, the plot shows good agreement between the experiment and the numerical model and a slight discrepancy is only visible for the reflected waves.

Similarly, Figure 7(d) refers to the results relative to the piezo rod. The figure shows that the amplitude of the incident pulse measured by the bottom cylindrical sensor is slightly smaller than the numerical prediction and the amplitudes of the reflected pulses are smaller than the corresponding numerical predictions. One possible reason is that the numerical model may underestimate the attenuation at the bead-cylinder interface, when the effect of possible static friction between the cylinder and the inner wall of the tube is ignored.

Finally, Figure 7(f) shows the experimental force profiles measured by the MsSs and the corresponding numerical result. By integrating the signal in Figure 7(e) and multiplying the integral with the calibration coefficient *K* listed in Table 1, Figure 7(f) is obtained. The presence of the magnetically induced precompresson reduces the attenuation and increases the pulse's velocity, thus reducing the TOF. The figure rveals the presence of a trough preceding the arrival of the main peak at both sensors. The origin of this response is not fully understood yet but we speculate that it might be associated with the presence of the permanent magnetic field. Nonetheless, the ability of the MsS to capture the presence and characteristics of HNSWs is evident. Overall Figure 7(b,d,f) shows very good agreement between the numerical and the experimental results relative to the incident pulse. There is some noticeable discrepancy in the time of arrival and amplitude of the reflected wave. This is likely due to the modeling of the rigid wall at the base of the chain.

To assess and compare the capability of the novel transducers to measure the characteristics of the HNSW propagating in a straight chain of particles, Tables 2 and 3 are presented. Table 2 summarizes the numerical and experimental values of the TOF and AR associated with the propagating pulse. The experimental values represent the average and the standard deviation (std) of the 500 measurements. The small variation between numerical and experimental values suggest that the two novel transducers perform equally well to the sensor bead. The small standard deviation also remarks the repeatability of the sensing systems. The speed of the incident and reflected solitary pulses are presented in Table 3. The small differences between numerical and experimental results indicate that all three types of sensors are able to measure the wave speed accurately. The smallest standard deviation, which was found for the magnetostrictive transducer, indicates the highest degree of repeatability of the setup. The speed of the solitary wave depends upon the setup. This is not surprising as the the solitary waves exhibit unique physical properties when compared to linear elastic waves. One of these properties is the dependency of the speed to the pulse's amplitude and to the level of precompression in the chain. Thus, the wave speed measured by the MsSs was the largest because they induce precompression magnetically. The speed measured by the piezo rods was the smallest as the presence of the solid rod diminishes the amplitude of the traveling pulse.

## 6. Discussion and Conclusions

In this paper we investigated numerically and experimentally three sensing systems for the measurements of highly nonlinear solitary pulses propagating in a chain of spherical particles. The transducers were a conventional pair of instrumented beads, and two novel designs based on the utilization of a pair of piezo rods, and a pair of coils. The latter aimed at exploiting the magnetostrictive properties of the particles.

We compared the experimental results to the numerical predictions obtained by means of a discrete particle model. We found that the two novel designs performed equally well when compared to the conventional sensor beads, which require micromachining and must not be allowed to rotate in order to keep their sensitivity constant. From this study the following consideration can be made. Owing to its geometry the piezoelectric cylinder is not prone to rotation and does not require the machining of half-particles. However it may originate unwanted secondary pulses that trails the incident wave and attenuates the amplitude of the incident pulse. To prevent this problem, the cylindrical sensor should have the same mass of the other particles composing the chain, and the sphere-cylinder contact stiffness should be the same as the sphere-sphere contact stiffness. Based on the contact mechanics theory, in order to have same contact stiffness, the elastic modulus of the cylinder should be approximately equal to 55% of the particle material's elastic modulus [11], which may not be feasible in practice for piezoelectric material. Moreover the wiring of the cylinder may be too brittle and therefore prone to rupture. Conversely, the use of coils has multi-fold advantage: it can be mounted outside the chain; it is noncontact; is can slide at convenience at any position along the chain. However, this design can be used only when the particles composing the chain are sensitive to magnetostriction, *i.e.*, they are able to convert magnetic energy into mechanical energy and vice versa. In fact, the magnetostrictive sensors exploit the coupling between the dynamic deformation of the magnetostrictive material to which they are applied and the variation of magnetic field surrounding the material. As confirmed by the empirical results, the voltage output is proportional to the dynamic deformation caused by the passage of the solitary wave pulse (Equation (5)). Future studies may look at improving the design of the magnetostrictive transducers and at investigating the response of the novel sensing system design in presence of much smaller and much larger crystals.

## Figures and Tables

**Table 1.** Calibration coefficients adopted in this study.

	**Calibration coefficient *K***	**Relative standard deviation (%)**	**Unit of *K***
Bead sensor top	2.072	4.71	N/V
Bead sensor bottom	2.160	4.31	N/V
Cylindrical sensor top	0.632	4.00	N/V
Cylindrical sensor bottom	0.557	4.01	N/V
MSS top	0.131	1.44	N/V·sec
MSS bottom	0.156	1.56	N/V·sec

**Table 2.** Experimental and numerical time-of-flight and amplitude ratio.

**Sensor Type**	**Sensor Position**	**Time of flight (microsec)**				**AR**			
	
		**Numerical**	**Experimental**		**Difference (%)**	**Numerical**	**Experimental**		**Difference (%)**
			**Mean**	**Std**			**Mean**	**Std**	
Bead	Top	318.7	309.8	2.6	2.79	0.57	0.55	0.04	3.51
	Bottom	227.5	219.9	1.6	3.34	0.68	0.73	0.02	7.35
Cylindrical	Top	341.0	337.0	5.3	1.17	0.42	0.37	0.03	11.9
	Bottom	234.9	228.4	3.0	2.77	0.68	0.60	0.07	11.8
MSS	Top	298.8	287.3	0.9	3.85	0.70	0.69	0.02	1.43
	Bottom	217.6	207.0	0.7	4.87	0.79	0.86	0.01	8.86

**Table 3.** Experimental and numerical speed of the incident and reflected HNSW pulse.

**Sensor Type**	**Type of Wave**	**Numerical (m/s)**	**Experimental (m/s)**		**Difference (%)**
			**mean**	**std**	
Bead	Incident	540.0	547.0	13.1	1.30
	Reflected	505.6	514.1	16.0	1.68
Cylindrical	Incident	486.3	512.0	14.6	5.28
	Reflected	458.7	422.9	38.6	7.80
MSS	Incident	596.8	602.1	1.75	0.89
	Reflected	576.6	584.8	3.36	1.42
